# Dietary phytogenic inclusion level affects performance and expression of heat shock, cytoprotective, inflammatory and apoptotic genes in the duodenum and the liver of cyclic heat-challenged broilers

**DOI:** 10.1016/j.psj.2025.105348

**Published:** 2025-05-26

**Authors:** Eugenia Iliopoulou, Ioannis Brouklogiannis, Vasileios V. Paraskeuas, Eirini Griela, Evangelos C. Anagnostopoulos, George Kefalas, Konstantinos C. Mountzouris

**Affiliations:** a Laboratory of Nutritional Physiology and Feeding, Department of Animal Science, School of Animal Biosciences, Agricultural University of Athens, Iera Odos 75, Athens 11855, Greece; b Feed Innovations & Technologies P.C., a spin-off company of the Agricultural University of Athens, Matsa 10, Kifisia 14665, Greece; c Nuevo SA, Schimatari, Viotia, Greece

**Keywords:** Poultry, Heat stress, Botanical, Nutrigenomic, Homeostasis

## Abstract

This study was conducted to investigate the inclusion level effect of a phytogenic blend (PB) on performance and the expression of genes relevant to heat shock response, detoxification, antioxidant capacity, inflammation and apoptosis in the duodenum and the liver of broilers under heat challenge conditions. The PB consisted of bioactive substances derived from oregano, thyme and olive oil polyphenols. Depending on PB inclusion level (i.e., 0, 500, 1,000, 1,500 and 2000 mg/kg diet) in the basal diet, 490 male broilers Ross 308, 1-d-old were assigned into 5 treatments: CON, PB500, PB1000, PB1500, and PB2000, with 7 replicates of 14 broilers, each. Birds were reared for 35 days under thermoneutral conditions and then subjected to 7 h cyclic heat stress (32 ± 1°C; RH 55 ± 5 %) for 5 consecutive days. Performance responses were closely monitored per growth phase (starter 1-10d, grower 11-24, finisher 25-40) and overall. At the end of the experiment (d40), duodenal and liver samples from 7 broilers per treatment were appropriately collected, snap frozen and subsequently stored at -80°C until gene expression analysis. Results revealed that PB inclusion level, improved (*P* < 0.05) Feed Conversion Ratio, Protein Efficiency Ratio, Energy Efficiency Ratio and the Nutritional Efficiency in a quadratic manner, compared to CON. At the molecular level, PB inclusion down-regulated (*P* < 0.05) most of the heat shock and inflammation-related genes, both in the duodenum and liver, in a linear manner with PB inclusion level. In addition, PΒ cytoprotective capacity was demonstrated via beneficial changes (*P* < 0.05) seen for the majority of the antioxidant and detoxification-related genes assessed, with the PB1500 displaying most significant differences, compared to CON. Conclusively, in heat challenged broilers, the multi-pathway nutrigenomic analysis provided new mechanistic support behind the improved broiler performance responses with PB inclusion.

## Introduction

Heat stress (HS) poses a significant challenge to all agricultural sectors, particularly poultry farming. More specifically, in intensive poultry production, broiler chickens are bred for rapid growth and high productivity and have reduced tolerance to high temperatures due to increased metabolic activity (Nawab et al., 2018). Therefore, chickens are vulnerable to the harmful effects of heat stress (Orlowski et al., 2018; Greene et al., 2021) that disturb cellular homeostasis and could lead to protein oxidation, lipid peroxidation, nucleic acid damage, apoptosis and inflammation (Song et al., 2019; Liu et al., 2021; Chen et al., 2024). Moreover, HS may have adverse effects on the broiler productive responses such as feed intake, growth performance (Quinteiro-Filho et al., 2010, 2012; Liu et al., 2022), as well as on meat quality (Zaboli et al., 2019).

The intestine plays a vital role in the digestion and absorption of essential nutrients and minerals (Zhang et al., 2017). Several studies have shown that heat stress negatively impacts intestinal homeostatic functions (Shi et al., 2019; Ayo and Ogbuagu, 2021) and compromises barrier integrity (Wu et al., 2018), which could lead to local intestinal inflammation (Tang et al., 2021) resulting in apoptosis. Simultaneously, the liver is the primary organ responsible for redox regulation and (Ma et al., 2019) detoxification (Zhang et al., 2017) and is essential for maintaining metabolic balance. It has been reported that heat stress impaired liver function (Chen et al., 2021) and triggered the production of Reactive Oxygen Species (ROS), further exacerbating oxidative damage and inflammation (Akbarian et al., 2016). Heat stress drives a cascade of reactions in both intestinal and hepatic tissues, disrupting homeostasis and inhibiting cellular functions throughout the organism. The aforementioned impact on these organs highlights the importance of developing effective strategies to mitigate the detrimental effects of HS on poultry health and productivity. In light of these challenges, alternative nutritional strategies are being researched. In this sense, phytogenics are being explored for their potential to mitigate heat stress (Greene et al., 2021; Mountzouris and Brouklogiannis, 2024). Phytogenics, which generally include aromatic plants, medicinal herbs, and spices, have triggered significant attention for their potential to improve animal performance and health. Phytogenic bioactive compounds are known for their antioxidant (Mountzouris et al., 2020), anti-inflammatory (Griela and Mountzouris, 2023), and cytoprotective properties (Brouklogiannis et al., 2023) in poultry, thus making them promising candidates for alleviating the negative effects of heat stress. Main factors affecting phytogenic efficiency are phytogenic composition and dietary inclusion levels (Amad et al., 2011; Pirgozliev et al., 2019; Anagnostopoulos and Mountzouris, 2025).

Despite the growing body of studies researching phytogenic applications in animal nutrition under heat stress conditions, limited research has explored collectively phytogenic effects on modulating heat shock, detoxification, antioxidant capacity, inflammation, and apoptotic biomarkers in broiler tissues.

Therefore, the present study aimed to evaluate the dietary inclusion level effects of a natural phytogenic blend (PB) on broiler performance and critical molecular biomarkers related to heat shock response, detoxification response, antioxidant capacity, inflammatory response and apoptosis during cyclic heat stress in broiler chickens. By investigating these parameters, this study aimed to provide new insights into the potential benefits of phytogenics in mitigating the adverse effects of heat challenge and enhancing broiler health and productivity.

## Materials and methods

### Ethics approval

All the procedures and practices used in this study regarding the care and use of animals for research purposes complied with the European Union Directives on the protection of animals used for scientific purposes (EC 43/2007; EU 63/2010). The experimental protocol was approved by the Bioethics Committee of the Agricultural University of Athens (Protocol number: 25/020523) and the competent Greek National authority Department of Agriculture and Veterinary Policy, General Directorate of Agriculture, Economy, Veterinary and Fisheries (Protocol number: 548007/080523).

### Birds, diets and experimental design

Day-old Ross 308 male broiler chicks (*n* = 490) were obtained from a commercial hatchery (Penelope Farm P.C., Viotia, Greece) and were vaccinated for Gumboro, Infectious Bronchitis and Newcastle disease. Upon arrival at the experimental facility, broilers were weighed and divided into the following five experimental treatments, depending on the PB inclusion level in the basal diet (BD): (i) control (CON), BD with no PB supplementation; (ii) PB500, BD containing 500 mg PB/kg diet; (iii) PB1000, BD containing 1000 mg PB/kg diet; (iv) PB1500, BD containing 1500 mg PP/kg diet; and (v) PB2000, BD containing 2000 mg PB/kg diet. Each treatment had 7 replicates with 14 broilers in a floor pen (1 m^2^) with rice-hull used as litter. The PB had olive oil polyphenols, carvacrol and thymol among its main bioactive components (NuPhoria®; Nuevo SA, Greece). The PB inclusion levels were chosen based on the total bioactive content of the PB and literature data, including previous experience by our research group including laying hens (Anagnostopoulos and Mountzouris, 2025; Brouklogiannis and Mountzouris, 2025). Throughout the experiment, diet and water were available *ad libitum*. The basal diets were formulated according to a three-growth stage feeding plan (i.e., starter, grower and finisher) and were typically based on maize and soybean meal. The calculated chemical composition per kilogram of the basal diets was as follows: starter (d1-10) (apparent metabolizable energy corrected for nitrogen [AMEn] 12.55 MJ, crude protein 230 g, lysine 14.4 g, calcium 9.6 g, and available phosphorus 4.8 g), grower (d11-24) (AMEn 12.97 MJ, crude protein 215 g, lysine 12.9 g, calcium 8.7 g, and available phosphorus 4.4 g) and finisher (d25-40) (AMEn 13.39 MJ, crude protein 195 g, lysine 11.5 g, calcium 7.8 g, and available phosphorus 3.9 g). Coccidiostat (Maxiban G160, Elanco Animal Health, Elli Lilly and Company, Clinton, Indiana, US) was included in all diets. The temperature was maintained in thermoneutral conditions, according to breeding manual (Aviagen Ross 308 FF Management Handbook, 2016), from day 1 to 35. On day 36 broilers were subjected to 7 h cyclic heat stress from 10:00 until 17:00 (32 ± 1°C; RH 55 ± 5 %) for 5 consecutive days.

### Broilers performance

Broilers were weighed per pen on starter (d1-10), grower (d11-21) and finisher (d22-40) phases. Average body weight (BW), body weight gain (BWG), feed intake (FI), feed conversion ratio (FCR), protein (PER) and energy (EER) efficiency ratio, European production efficiency factor (EPEF) and nutritional efficiency (NEF) were evaluated for the entire duration of the experiment (40 days). FCR was calculated based on FI and average BWG of each pen. The PER was calculated as grams of BWG per gram of protein intake, whereas the EER was calculated as grams of BWG × 100/total ME intake. EPEF was calculated based on viability, body weight, age and FCR (EPEF = (Livability (%) x live weight (kg)) / (age (days) x FCR) x 100). The NEF (€/kg feed) was calculated based on the purchase prices of raw materials according to the recipe structure. Knowing FI on growth periods, BWG, FCR and compound feed cost was calculated the feed cost/kg live weight (€/kg BW).

### Tissue sampling for subsequent analyses

At the end of the trial (d40) 7 broilers per treatment were randomly selected anaesthetized (EC 1099/2009) and euthanized by severing the jugular vein. Subsequently, the duodenum and liver were carefully excised aseptically, snap-frozen in liquid nitrogen, and subsequently stored at −80°C for further analyses.

### RNA isolation and reverse transcription to cDNA

For the determination of target gene expression, total RNA from duodenum and liver samples was isolated by using NucleoZOL Reagent (Macherey-Nagel GmbH & Co. KG, Duren, DE), according to the manufacturer’s protocol. Subsequently, RNA quality and quantity were assessed by spectrophotometry (NanoDrop™ One/One^C^ Microvolume UV-Vis Spectrophotometer, Thermo Fisher Scientific, Waltham, US). DNase treatment followed in duodenum and liver samples. In particular 10 mg of RNA were diluted with 1 U of DNase I (M0303, New England Biolabs Inc, Ipswich, UK) and 10 μL of 10x DNAse buffer to a final volume of 100 μL with the addition of DEPC water for 20 min at 37°C. Prior to DNAse inactivation at 75°C for 10 min, 1 μL of 0.5 mol/L EDTA was added to protect RNA from being degraded during enzyme inactivation. RNA integrity was assessed by agarose gel electrophoresis. For cDNA preparation, 750 ng of total RNA from each sample were reverse transcribed to cDNA by PrimeScript RT Reagent Kit (Perfect Real Time, Takara Bio Inc., ShigaKen, JP) following the manufacturer’s guidelines. All cDNAs were afterwards stored at −20°C.

### Quantitative real-time PCR analysis

The following *Gallus gallus* genes were examined: heat shock factor 1, 3, 4 (*HSF1, HSF3, HSF4*), heat shock protein 27, 47, 60, 70, 90, 100 (*HSP27, HSP47, HSP60, HSP70, HSP90, HSP100*), aryl hydrocarbon receptor 1 (*Ahr1*), aryl hydrocarbon receptor nuclear translocator (*ARNT*), cytochrome P450 1A1 (*CYP1A1*), cytochrome P450 1A2 (*CYP1A2*), cytochrome P450 1B1 (*CYP1B1*), nuclear factor erythroid-derived 2-like 2 (*Nrf2*), kelch like ECH associated protein 1 (*Keap1*), catalase (*CAT*), superoxide dismutase 1 (*SOD1*), glutathione peroxidase 2, 7 (*GPX2, GPX7*), glutathione S-transferase alpha 2 (*GSTA2*), NAD(P)H quinone dehydrogenase 1 (*NQO1*), heme oxygenase 1 (*HMOX1*), toll-like receptor 2 family member B (*TLR2B*), toll-like receptor 3 (*TLR3*), toll-like receptor 4 (*TLR4*), toll-like receptor adaptor molecule 1 (*TRIF*), myeloid differentiation primary response 88 (*MyD88*), interferon regulatory factor 3 (*IRF3*), nuclear factor kappa B subunit 1 (*NF-kB1*), interleukin 6 (*IL6*), interleukin 8 (*IL8*), interferon-beta (*IFNW*), lipopolysaccharide-induced TNF factor (*LITAF*), transforming growth factor beta 1 (*TGFB1*), interleukin 1β (*IL1B*), cyclooxygenase 2 (*COX2*), Fas cell surface death receptor (*APO-1/FAS*), mitogen-activated protein kinase 9 (*MAPK9*), conserved helix-loop-helix ubiquitous kinase (*CHUK*), Fos proto-oncogene, AP-1 transcription factor subunit (*FOS*), Jun proto-oncogene AP-1 transcription factor subunit (*JUN*), caspase 3, 8 (*CASP3, CASP8*), Fas associated via death domain (*FADD*), and B-cell lymphoma 2 (*BCL2*).

Suitable primers were designed using the GenBank sequences deposited on the National Center for Biotechnology Information and US National Library of Medicine (NCBI) shown in Table 1. Primers were checked using the PRIMER BLAST algorithm for *Gallus gallus* mRNA databases to ensure that there was a unique amplicon. Real-time PCR was accomplished in 96 well microplates with a SaCycler-96 Real-Time PCR System (Sacace Biotechnologies s.r.l., Como, IT) and FastGene IC Green qPCR universal mix (Nippon Genetics, Tokyo, JP). Each reaction included 12.5 ng RNA equivalents as well as 200 nmol/L of forward and reverse primers for each gene. The reactions were incubated at 95°C for 3 min, followed by 40 cycles of 95°C for 5 s 59.5 to 62°C (depends on the target gene) for 20 s, 72°C for 33 s. This was followed by a melt curve analysis to regulate the reaction specificity. Each sample was determined in duplicates. Relative expression ratios of target genes were calculated according to Pfaffl. (2001) adapted for the multi-reference genes normalization procedure according to Hellemans et al. (2008) using *GAPDH* (glyceraldehyde-3-phosphate dehydrogenase) and *ACTB* (actin beta) as reference genes. In particular, the expression of each target gene was normalized to the geometric mean of two validated housekeeping genes: *GAPDH* and *ACTB*. These reference genes were selected for their stable expression across experimental conditions.

**Table 1:** Oligonucleotide primers used for gene expression of selected targets by quantitative real time PCR.

Target	Primer sequence (5′−3′)	Annealing temperature (⁰C)	PCR product size (bp)	GenBank
GAPDH	F: ACTTTGGCATTGTGGAGGGT	59.5	131	NM_204305.1
	R: GGACGCTGGGATGATGTTCT			
ACTB	F: CACAGATCATGTTTGAGACCTT	60	101	NM_205518.1
	R: CATCACAATACCAGTGGTACG			
Heat Shock Response pathway				
HSF1	F: GCTTCGCCAACTACATCGAC	59.5	145	NM_001305256.1
	R:TCAAGATCAAGGCGTGCCAG			
HSF3	F:GCCGGAATGGCGAGAACTT	59.5	108	NM_001305041
	R:TGCCACTTCTTTCCACAGGG			
HSF4	F: AGAACCCACCTCCTGTTTGC	59.5	193	NM_001172374.3
	R: TGGGATGTCGGGTTCATTGG			
HSP27 (HSPB1)	F: ATTGGTACAAGTGGCCCAGC	59.5	195	NM_205290.2
	R: CAGGAGCAAAGTGGTTGACG			
HSP47	F: AGGTGGCTTTGGGATGTCAG	60	263	XM_040665757.2
	R: CAGCAGAGGAGTCCCTTTGC			
HSP60	F: ATGATGCGATGCTTGGGGAA	60	268	NM_001012916.3
	R: CAGAGCCGTTCTCACAACCT			
HSP70	F: ATGCTAATGGTATCCTGAACG	60	145	NM_001006685.1
	R: TCCTCTGCTTTGTATTTCTCTG			
HSP90	F: CACGATCGCACTCTGACCAT	60	196	NM_001109785.1
	R: CTGTCACCTTCTCCGCAACA			
HSP100	F: GTGGAAGCAGAGCTGGAGAA	59.5	134	XM_046904904.1
	R: GCATACAGCACTACAGAGCC			
AhR pathway				
AhR	F: TTTAGTGTGGCAGGTGGATT	60	200	NM_204118.2
	R: CCTTGTGCCAATGATGCTATTTG			
ARNT	F: GAGACCAAGGCCCCAACTAC	62	140	NM_204200.1
	R: TCGGGTGCCTCTTTCTTTCC			
CYP1A1	F: GTGATGGAGGTGACCATCGG	62	165	NM_205147.1
	R: ACATTCGTAGCTGAACGCCA			
CYP1A2	F: CTGACCGTACACCACGCTT	62	75	NM_205146.2
	R: CTCGCCTGCACCATCACTTC			
CYP1B1	F: CAGTGACTCCGCATCCCAAA	62	132	XM_015283751.2
	R: CCATACGCTTACGGCAGGTT			
Keap1/Nrf2/ARE pathway				
Nrf2	F:AGACGCTTTCTTCAGGGGTAG	60	285	NM_205117.1
	R:AAAAACTTCACGCCTTGCCC			
Keap1	F: GGTTACGATGGGACGGATCA	62	135	XM_025145847.1
	R: CACGTAGATCTTGCCCTGGT			
CAT	F: ACCAAGTACTGCAAGGCGAA	60	245	NM_001031215
	R: TGAGGGTTCCTCTTCTGGCT			
SOD1	F: AGGGGGTCATCCACTTCC	60	122	NM_205064.1
	R: CCCATTTGTGTTGTCTCCAA			
GPX2	F: GAGCCCAACTTCACCCTGTT	62	75	NM_001277854.1
	R: CTTCAGGTAGGCGAAGACGG			
GPX7	F: GGCTCGGTGTCGTTAGTTGT	60	139	NM_001163245.1
	R: GCCCAAACTGATTGCATGGG			
GSTA2	F: GCCTGACTTCAGTCCTTGGT	60	138	NM_001001776.1
	R: CCACCGAATTGACTCCATCT			
NQO1	F: GAGCGAAGTTCAGCCCAGT	60.5	150	NM_001277619.1
	R: ATGGCGTGGTTGAAAGAGGT			
HMOX1	F: ACACCCGCTATTTGGGAGAC	62	134	NM_205344.1
	R: GAACTTGGTGGCGTTGGAGA			
TLR to NF-κB signaling pathway				
TLR2B	F: CTTGGAGATCAGAGTTTGGA	62	238	NM_001161650.1
	R: ATTTGGGAATTTGAGTGCTG			
TLR3	F: GCTTGGTTTGCTAGTTGGCT	59.5	93	NM_001011691.3
	R: ACCGTGATATTTAGGCGGGG			
TLR4	F:GTCTCTCCTTCCTTACCTGCTGTTC	64.5	187	NM_001030693.1
	R: AGGAGGAGAAAGACAGGGTAGGTG			
TRIF	F: TCAGCCATTCTCCGTCCTCTTC	62	339	NM_001081506.1
	R:GGTCAGCAGAAGGATAAGGAAAGC			
MyD88	F: AATGGACACTGAGCTCTGCC	60	126	NM_001030962.3
	R: CAAACCCGATCTGTGGGACA			
IRF3	F: GAGGATCCGGCCAAATGGAA	60	212	NM_205372.1
	R: GCCAAATCGTGGTGGTTGAG			
NFkB1	F: GAAGGAATCGTACCGGGAACA	59	131	NM_205134.1
	R:CTCAGAGGGCCTTGTGACAGTA			
IL8	F: CAGGTGACACCCGGAAGAAA	61	117	NM_205018.1
	R: CTGAACGTGCCTGAGCCATA			
IFNW	F: CCTCAACCAGATCCAGCATTAC	60.5	167	NM_001024836.1
	R: CCCAGGTACAAGCACTGTAGTT			
LITAF	F: GAGCAGGGCTGACACGGAT	60	149	NM_204267.1
	R: GCACAAAAGAGCTGATGGCAG			
TGFB1	F: GGTTATATGGCCAACTTCTGCAT	60	102	JQ423909.1
	R: CCCCGGGTTGTGTTGGT			
IL6	F: AAATCCCTCCTCGCCAATCT	59	106	NM_204628.1
	R: CCCTCACGGTCTTCTCCATAAA			
IL1B	F: CAGGTGACACCCGGAAGAAA	59.5	207	NM_204524
	R: CTGAACGTGCCTGAGCCATA			
iNOS				
COX2	F: TGTCCTTTCACTGCTTTCCAT	60	232	NM_001167718
	R: TTCCATTGCTGTGTTTGAGGT			
MAPK - apoptosis pathway				
CHUK (IKK)	F: TTCACTGGTAAGCTCCAGCC	60	199	NM_001012904.1
	R: TTCTCTTGCCTCCTGCAACA			
FOS	F: GCCGACATGATGTACCAGGG	62	101	NM_205508.1
	R: GACGGGTAGTAGGTGAGGCT			
JUN	F: CCTCCCCTGTCCCCTATTGA	61.5	99	NM_001031289.1
	R: CCTTTTCCGGCATTTGGACG			
FAS	F: TGGTCAGTGCTGCACGATAA	59.5	381	NM_001199487
(APO-1)	R: CAACCTCCAAACCGAGTGCT			
MAPK9	F: TTGGACTGGCAAGAACAGCG	62	86	NM_205095.1
	R: AAGGATAACCTCTGGCGCTC			
CASP3	F: TGGTGGAGGTGGAGGAGC	60	110	NM_204725.2
	R: CCTGAGCGTGGTCCATCTTT			
CASP8	F: TGTTGATGCCAGCCTTCCCTG	60	126	NM_204592.4
	R: TTGCCAATAACCCACCCCG			
FADD	F:AGGGGAAAGATGCGAAGGTG	59.5	146	XM_040673265
	R: ACCCGTGGTCTGAGTGTTTT			
BCL2	F: GCTGCTTTACTCTTGGGGGT	59.5	128	NM_205339.3
	R: CTTCAGCACTATCTCGCGGT			

^1^GAPDH = glyceraldehyde 3-phosphate dehydrogenase; ACTB = actin beta; HSF1 = heat shock factor 1; HSF3 = heat shock factor 3; HSF4 = heat shock factor 4; HSP27 = heat shock protein27; HSP47 = heat shock protein47; HSP60 = heat shock protein 60; HSP70 = heat shock protein 70; HSP90 = heat shock protein 90 family class A member 1; HSP100 = heat shock protein 100; AhR1 = aryl hydrocarbon receptor 1; ARNT = aryl hydrocarbon receptor nuclear translocator; CYP1A1 = cytochrome P450 1A1; CYP1A2 = cytochrome P450 1A2; CYP1B1 = cytochrome P450 1B1; Nrf2 = nuclear factor; erythroid 2-like 2; Keap1 = kelch-like ECH-associated protein 1; CAT = catalase; SOD1 = superoxide dismutase 1; GPX2,7 = glutathione peroxidase 2, 7; GSTA2 = glutathione S-transferase alpha 2; NQO1 = NAD(P)H quinone dehydrogenase 1; HMOX1 = heme oxygenase 1; TLR2B = toll-like receptor 2B; TLR3 = toll-like receptor 3; TLR4 = toll-like receptor 4; TRIF = toll-like receptor adaptor molecule 1; MyD88 = myeloid differentiation primary response 88; IRF3 = interferon regulatory factor 3; NF-κB1 = nuclear factor kappa B subunit 1; IL8 = interleukin 8; IFNW = interferon beta; LITAF = lipopolysaccharide-induced TNF Factor; TGFB1 = transforming growth factor beta 1; IL6 = interleukin 6; IL1B = interleukin 1β; iNOS = nitric oxide synthase; COX2 = cyclooxygenase-2; CHUK = conserved helix-loop-helix ubiquitous kinase; FOS = Fos proto-oncogene, AP-1 transcription factor subunit; JUN = Jun proto-oncogene AP-1 transcription factor subunit; FAS/APO-1 = Fas cell surface death receptor; MAPK9 = mitogen-activated protein kinase 9; CASP3,8 = caspase 3,8; FADD = FAS-associated death domain protein; Bc-l2 = *B*-cell lymphoma 2.

^2^F: Forward, R: Reverse.

### Statistical analysis

Experimental data were initially checked for normality using the Kolmogorov–Smirnov test and found to be normally distributed. Data were subsequently analyzed with the general linear model (GLM)−ANOVA procedure using the SPSS for Windows statistical package program, version 27 (SPSS Inc., Chicago, IL). Statistically significant effects were further analyzed, and means were compared using Tukey’s honest significant difference (HSD) multiple comparison procedure. Statistical significance was determined at *P* ≤ 0.05. Linear (lin) and quadratic (quad) response patterns to dietary PB inclusion level were studied using polynomial contrasts.

## Results

### Broilers performance responses

The overall broiler performance responses are shown in Table 2. There were no significant differences (*P* > 0.05) in BW, BWG, FI, and EPEF between the experimental treatments. Compared to the CON, the FCR (*P* < 0.012), PER (*P* < 0.007), EER (*P* < 0.005) and NEF (*P* < 0.019) were improved with PB inclusion. Specifically, FCR (*P* *<* 0.012), PER (*P* < 0.007), EER (*P* < 0.005) and NEF (*P* < 0.0019) were improved in PB1000 and PB1500 compared to CON. In addition, FCR (*P_quad_* < 0.002), NEF (*P_quad_* < 0.001), PER (*P_quad_* < 0.002), and EER (*P_quad_* < 0.001) displayed a quadratic pattern of decrease.

**Table 2:** Effect of dietary PB inclusion on overall (d1-40) broiler growth performance responses.

Components^4^	Experimental Treatments^1^						Statistics^2^		
	CON	PB500	PB1000	PB1500	PB2000	SEM^3^	*P_ANOVA_*	*P_LINEAR_*	*P_QUADRATIC_*
Body weight	2326.5	2389.3	2408.7	2348.4	2387.4	60.14	0.652	0.555	0.433
Body weight gain	2288.7	2351.4	2370.8	2310.6	2349.5	60.13	0.652	0.555	0.433
Feed Intake	3563.8	3571.1	3512.3	3423.0	3574.2	83.97	0.353	0.506	0.260
FCR	1.56^a^	1.52^ab^	1.48^b^	1.48^b^	1.52^ab^	0.021	0.012	0.051	0.002
PER	308.6^A^	301.2^AB^	293.5^B^	294.0^B^	301.5^AB^	4.02	0.007	0.026	0.002
EER	20.6^A^	20.1^AB^	19.6^B^	19.5^B^	20.1^AB^	0.26	0.005	0.023	0.001
EPEF	352.8	381.9	377.7	379.7	382.2	24.02	0.710	0.305	0.469
NEF	0.88^ab^	0.86^ab^	0.85^b^	0.86^ab^	0.89^a^	0.012	0.019	0.765	0.001

1 PB Inclusion (CON = 0 mg/kg, PB500 = 500 mg/kg, PB1000 = 1000 mg/kg, PB1500 = 1500 mg/kg, and PB2000 = 2000 mg/kg of diet). Data represent treatment means from n = 7 pens of 14 broiler chicks per pen.

2 Means with different superscripts (a, b or A, B) within the same row differ significantly (*P* < 0.05 or 0.01).

3 Standard error of the means.

4 FCR= feed conversion ratio; PER= protein conversion ratio; EER= energy conversion ratio; EPEF= European production efficiency factor; NEF= nutritional efficiency factor.

### Relative expression of the critical genes studied in the duodenum and liver

The nutrigenomic analysis results are presented per each of the homeostasis related pathway studied

### Heat shock response pathway

The relative expression of Heat Shock Response pathway-related genes (*HSF1, HSF3, HSF4, HSP27, HSP47, HSP60, HSP70, HSP90* and *HSP100*) in the duodenum and the liver at day 40 of the experiment were presented in Table 3. The expression of *HSF1* (*P* < 0.001), *HSF4* (*P* < 0.001), *HSP47* (*P* = 0.002), *HSP70* (*P* < 0.001), *HSP90* (*P* = 0.004), and *HSP100* (*P* < 0.001) differed between the experimental treatments. Increasing PB inclusion level under heat challenge conditions resulted in linear patterns of decrease for *HSF1* (*P_lin_ <* 0.001), *HSF3* (*P_lin_* = 0.016), *HSF4* (*P_lin_* < 0.001), *HSP47* (*P_lin_* < 0.001), *HSP70* (*P_lin_* < 0.001), *HSP90* (*P_lin_* < 0.001), and *HSP100* (*P_lin_* < 0.001). *HSF1* (*P_quad_* = 0.001), *HSF4* (*P_quad_* = 0.025), and *HSP100* (*P_quad_* = 0.046) expression displayed quadratic patterns of decrease. Compared to CON, the relative expression levels of *HSF1, HSF4, HSP47, HSP70, HSP90* and *HSP100* were lowest for PB2000. At the liver, the expression of *HSF1* (*P* < 0.001), *HSF4* (*P* = 0.001), *HSP27* (*P* < 0.001), *HSP47* (*P* = 0.008), *HSP70* (*P* < 0.001), *HSP90* (*P* < 0.001), and *HSP100* (*P* < 0.001) differed between the experimental treatments. Increasing PB inclusion level under heat challenge conditions resulted in linear patterns of decrease for *HSF1* (*P_lin_* < 0.001), *HSF3* (*P_lin_* = 0.003), *HSF4* (*P_lin_* < 0.001), *HSP27* (*P_lin_* < 0.001), *HSP47* (*P_lin_* = 0.001), *HSP70* (*P_lin_* < 0.001), *HSP90* (*P_lin_* < 0.001), and *HSP100* (*P_lin_* < 0.001). The expression of *HSF4* (*P_quad_* = 0.001), and *HSP27* (*P_quad_* = 0.002) displayed quadratic patterns of decrease. Compared to CON, the relative expression levels of *HSF1, HSP27, HSP47, HSP70, HSP90* and *HSP100* were lowest for PB2000 under heat challenge conditions. Moreover, *HSF4* was lowest for PB1500 compared to the control treatment.

**Table 3:** Relative gene expression of heat shock response-related genes in broilers at day 40 of the experiment.

	Genes^4^	Treatments^1^						Statistics^2^		
		CON	PB500	PB1000	PB1500	PB2000	SEM^3^	*P_ANOVA_*	*P_LINEAR_*	*P_QUADRATIC_*
	HSF1	2.15^A^	0.98^B^	0.91^BC^	0.89^BC^	0.55^C^	0.168	<0.001	<0.001	0.001
	HSF3	1.47	0.98	1.04	0.90	0.84	0.235	0.086	0.016	0.295
	HSF4	1.88^A^	1.21^B^	0.99^B^	0.71^B^	0.69^B^	0.196	<0.001	<0.001	0.025
Duodenum	HSP27	1.02	0.79	1.35	1.23	0.88	0.268	0.224	0.780	0.211
	HSP47	1.47^A^	1.04^AB^	0.89^B^	1.02^AB^	0.72^B^	0.169	0.002	<0.001	0.240
	HSP60	1.08	0.84	1.07	0.93	1.01	0.146	0.436	0.852	0.477
	HSP70	1.82^A^	1.24^AB^	1.00^B^	0.79^B^	0.64^B^	0.226	<0.001	<0.001	0.152
	HSP90	2.02^A^	1.38^AB^	1.02^B^	1.02^B^	0.58^B^	0.256	0.004	<0.001	0.361
	HSP100	2.48^A^	1.14^B^	1.28^B^	0.77^BC^	0.38^C^	0.227	<0.001	<0.001	0.046
	HSF1	1.72^A^	1.42^AB^	1.19^ABC^	0.72^BC^	0.48^C^	0.255	<0.001	<0.001	0.863
	HSF3	1.13^a^	1.00^abc^	1.06^ab^	0.86^bc^	0.82^c^	0.105	0.030	0.003	0.775
	HSF4	1.83^A^	1.34^AB^	0.73^B^	0.70^B^	1.09^B^	0.241	0.001	<0.001	0.001
Liver	HSP27	1.76^A^	1.18^B^	0.83^BC^	0.84^BC^	0.64^C^	0.126	<0.001	<0.001	0.002
	HSP47	1.94^A^	1.20^AB^	0.86^B^	0.92^B^	0.68^B^	0.342	0.008	0.001	0.134
	HSP60	1.07	1.09	1.00	1.04	1.07	0.203	0.993	0.910	0.796
	HSP70	1.82^A^	1.03^B^	1.11^B^	1.02^B^	0.77^B^	0.210	<0.001	<0.001	0.107
	HSP90	1.34^A^	1.06^AB^	0.92^B^	0.87^B^	0.75^B^	0.119	<0.001	<0.001	0.181
	HSP100	2.33^A^	1.91^A^	1.06^B^	0.68^BC^	0.30^C^	0.231	<0.001	<0.001	0.375

1 PB Inclusion (CON = 0 mg/kg, PB500 = 500 mg/kg, PB1000 = 1000 mg/kg, PB1500 = 1500 mg/kg, and PB2000 = 2000 mg/kg of diet). Data represent treatment means from n = 7 broiler tissue samples per treatment.

2 Means with different superscripts (a, b, c or A, B, C) within the same row differ significantly (*P* < 0.05 or 0.01).

3 Standard error of the means.

4 HSF1 = heat shock factor 1; HSF3 = heat shock factor 3; HSF4 = heat shock factor 4; HSP27 = heat shock protein27; HSP47 = heat shock protein47; HSP60 = heat shock protein 60; HSP70 = heat shock protein 70; HSP90 = heat shock protein 90 family class A member 1; HSP100 = heat shock protein 100.

### Detoxification AhR pathway

The expression levels of the detoxification AhR pathway (*AHR, ARNT, CYP1A1, CYP1A2* and *CYP1B1*) related genes in the duodenum and the liver at day 40 of the experiment are presented in Table 4. The expression of *AHR* (*P* = 0.003), *ARNT* (*P* = 0.011) and *CYP1A1* (*P* < 0.001) differed between the experimental treatments in the duodenum. Increasing PB inclusion level resulted in linear patterns of decrease for *AHR* (*P_lin_* < 0.001), *ARNT* (*P_lin_* <0.001) and *CYP1A1* (*P_lin_* = 0.002). *CYP1A1* (*P_quad_* = 0.023) and *CYP1A2* (*P_quad_* = 0.006) expression displayed quadratic patterns of decrease. Compared to CON, the relative expression level of *AHR* was the lowest for PB2000 under heat challenge conditions. *ARNT* was the lowest for PB1500 and *CYP1A1* was the lowest for PB1000 compared to CON. At the liver, the relative expression of *AHR* (*P* = 0.006), *ARTN* (*P* = 0.007), *CYP1A1* (*P* < 0.001) and *CYP1B1* (*P* = 0.003) differed between the experimental treatments. Polynomial contrast analysis showed that the expression of *ARNT* (*P_lin_* < 0.001), *CYP1A1* (*P_lin_* < 0.001) and *CYP1B1* (*P_lin_* = 0.010) displayed linear patterns of decrease with increasing PB inclusion level. In addition, PB inclusion level resulted in quadratic pattern of decrease for the *AHR* (*P_quad_* = 0.001), *ARNT* (*P_quad_* < 0.001), *CYP1A2* (*P_quad_* = 0.025) and *CYP1B1* (*P_quad_* = 0.011). Compared to control treatment, the relative expression of *CYP1A1* was lowest for PB2000 treatment, *ARNT* was lowest for PB1500 treatment, *AHR* and *CYP1B1* were the lowest for PB1000 treatment under heat challenge conditions.

**Table 4:** Relative gene expression of detoxification response-related genes in broilers at day 40 of the experiment.

	Genes^4^	Treatments^1^						Statistics^2^		
		CON	PB500	PB1000	PB1500	PB2000	SEM^3^	*P_ANOVA_*	*P_LINEAR_*	*P_QUADRATIC_*
Duodenum	AHR	1.52^A^	1.28^AB^	0.89^BC^	0.81^BC^	0.69^C^	0.165	0.003	<0.001	0.212
	ARNT	1.52^a^	1.30^ab^	0.87^ab^	0.65^b^	0.84^b^	0.228	0.011	<0.001	0.097
	CYP1A1	2.07^A^	1.68^AB^	0.61^B^	0.67^B^	1.03^AB^	0.412	<0.001	0.002	0.023
	CYP1A2	1.58	0.88	0.75	0.99	1.33	0.313	0.074	0.588	0.006
	CYP1B1	1.05	0.77	1.20	1.27	1.05	0.298	0.507	0.447	0.761
Liver	AHR	1.33^a^	0.81^b^	0.79^b^	0.96^ab^	1.08^ab^	0.148	0.006	0.292	0.001
	ARNT	2.71^a^	1.07^b^	0.72^b^	0.65^b^	0.78^b^	0.358	0.007	<0.001	<0.001
	CYP1A1	1.89^a^	1.24^ab^	1.15^abc^	0.75^bc^	0.46^c^	0.268	<0.001	<0.001	0.576
	CYP1A2	1.31	0.98	0.82	0.86	1.18	0.241	0.224	0.499	0.025
	CYP1B1	1.36^a^	0.85^b^	0.82^b^	0.98^ab^	0.87^b^	0.138	0.003	0.010	0.011

1 PB Inclusion (CON = 0 mg/kg, PB500 = 500 mg/kg, PB1000 = 1000 mg/kg, PB1500 = 1500 mg/kg, and PB2000 = 2000 mg/kg of diet). Data represent treatment means from n = 7 broiler tissue samples per treatment.

2 Means with different superscripts (a, b, c or A, B, C) within the same row differ significantly (*P* < 0.05 or 0.01).

3 Standard error of the means.

4 AhR1 = aryl hydrocarbon receptor 1; ARNT = aryl hydrocarbon receptor nuclear translocator; CYP1A1 = cytochrome P450 1A1; CYP1A2 = cytochrome P450 1A2; CYP1B1 = cytochrome P450 1B1.

### Antioxidant capacity Nrf2/ARE pathway

The expression levels of Nrf2 pathway-related genes (*NRF2, KEAP1, CAT, SOD, GPX2, GPX7, GSTA2, NQO1* and *HMOX1*) in the duodenum and the liver at day 40 of the experiment are presented in Table 5. The expression of *NRF2 (P* = 0.041), *KEAP1* (*P* = 0.010), *CAT* (*P* < 0.001), *SOD* (*P* = 0.002), *GPX7* (*P* = 0.011), *GSTA2* (*P* = 0.009), and *HMOX1* (*P* = 0.004) differed between the experimental treatments at the duodenum. Increasing PB inclusion level under heat challenge conditions resulted in linear patterns of increase for *NRF2* (*P_lin_* = 0.002), *CAT* (*P_lin_* < 0.001), *SOD* (*P_lin_* = 0.002), *GPX7* (*P_lin_* < 0.001), *GSTA2* (*P_lin_* = 0.001), *HMOX1* (*P_lin_* = 0.007), and decrease for *KEAP1* (*P_lin_* < 0.001). Moreover, *SOD* (*P_quad_* = 0.027) expression displayed quadratic patterns of increase. Compared to CON, the relative expression levels of *NRF2, CAT, GPX7* and *GSTA2* were highest for PB2000. In the case of *SOD, HMOX1* and *KEAP1* were lowest for PB1500 compared to CON under heat challenge conditions. At the liver, the expression of *KEAP1* (*P* = 0.037), *SOD* (*P* < 0.001), *GPX2* (*P* < 0.001), *GPX7* (*P* = 0.003) and *GSTA2* (*P* = 0.036) differed between the experimental treatments. Increasing PB inclusion level under heat challenge conditions resulted in linear patterns of increase for *SOD* (*P_lin_* < 0.001), *GPX7* (*P_lin_* < 0.001), *GSTA2* (*P_lin_* < 0.001), and decrease *KEAP1* (*P_lin_* = 0.004). *SOD* (*P_quad_* = 0.003) expression displayed quadratic patterns of increase. Compared to CON, the relative expression levels of *CAT, GPX7* and *GSTA2* were highest for PB2000. Additionally, *SOD, HMOX1* and *KEAP1* were lowest for PB1500 compared to CON.

**Table 5:** Relative gene expression of antioxidant capacity response-related genes in broilers at day 40 of the experiment.

	Genes^4^	Treatments^1^						Statistics^2^		
		CON	PB500	PB1000	PB1500	PB2000	SEM^3^	*P_ANOVA_*	*P_LINEAR_*	*P_QUADRATIC_*
Duodenum	NRF2	0.83^b^	0.88^b^	1.24^ab^	1.51^ab^	1.77^a^	0.339	0.041	0.002	0.715
	KEAP1	1.47^A^	1.30^AB^	1.00^AB^	0.72^B^	0.75^B^	0.205	0.010	<0.001	0.448
	CAT	0.79^C^	0.98^BC^	1.41^AB^	1.13^BC^	1.67^A^	0.184	<0.001	<0.001	0.984
	SOD	0.88^B^	1.08^B^	1.16^AB^	1.44^A^	1.16^AB^	0.121	0.002	0.002	0.027
	GPX2	0.81	1.13	1.35	1.39	1.39	0.227	0.071	0.009	0.198
	GPX7	0.76^b^	0.88^b^	1.20^ab^	1.21^ab^	1.56^a^	0.223	0.011	<0.001	0.808
	GSTA2	0.85^B^	0.99^AB^	1.23^AB^	1.14^AB^	1.34^A^	0.136	0.009	0.001	0.564
	NQO1	0.96	1.30	1.06	1.02	1.23	0.157	0.177	0.465	0.886
	HMOX1	0.70^B^	1.05^AB^	1.29^AB^	1.59^A^	1.39^AB^	0.298	0.004	0.007	0.204
Liver	NRF2	0.77	1.14	1.31	1.34	1.21	0.233	0.134	0.048	0.076
	KEAP1	1.36^a^	0.99^b^	0.82^b^	0.83^b^	0.92^b^	0.147	0.037	0.004	0.008
	CAT	0.88	1.33	1.50	1.31	1.50	0.271	0.161	0.053	0.222
	SOD	0.58^B^	1.32^A^	1.24^A^	1.41^A^	1.19^A^	0.200	<0.001	0.006	0.003
	GPX2	0.69^B^	1.44^AB^	1.91^A^	1.19^AB^	1.35^AB^	0.401	<0.001	0.236	0.033
	GPX7	0.78^B^	0.97^AB^	1.04^AB^	1.42^A^	1.36^A^	0.181	0.003	<0.001	0.736
	GSTA2	0.81^b^	1.05^ab^	0.99^b^	1.26^b^	1.50^a^	0.168	0.036	<0.001	0.465
	NQO1	0.86^b^	1.03^b^	1.00^b^	1.07^ab^	1.52^a^	0.161	0.036	0.001	0.135
	HMOX1	1.21	1.00	0.93	1.40	1.15	0.297	0.521	0.501	0.507

1 PB Inclusion (CON = 0 mg/kg, PB500 = 500 mg/kg, PB1000 = 1000 mg/kg, PB1500 = 1500 mg/kg, and PB2000 = 2000 mg/kg of diet). Data represent treatment means from n = 7 broiler tissue samples per treatment.

2 Means with different superscripts (a, b, c or A, B, C) within the same row differ significantly (*P* < 0.05 or 0.01).

3 Standard error of the means.

4 Nrf2 = nuclear factor; erythroid 2-like 2; Keap1 = kelch-like ECH-associated protein 1; CAT = catalase; SOD1 = superoxide dismutase 1; GPX2,7 glutathione peroxidase 2, 7; GSTA2 = glutathione S-transferase alpha 2; NQO1 = NAD(P)H quinone dehydrogenase 1; HMOX1 = heme oxygenase 1.

### Inflammatory response (TLR to NF-κΒ pathway)

The expression levels of TLR to NF-κΒ pathway-related genes (*TLR2, TLR4, MYD88, NFKB, TLR3, IRF3, TRIF, IFNW, TGF, LITAF, iNOS, IL1B, IL6, IL*8 and *COX2*) in the duodenum and the liver at day 40 of the experiment under heat challenge conditions are presented in Table 6. The expression of *TLR4* (*P* < 0.001), *TLR3* (*P* = 0.002), *IRF3* (*P* < 0.001), *TGF* (*P* = 0.010), *LITAF* (*P* = 0.001), *iNOS* (*P* < 0.001), *IL1B* (*P* = 0.003), *IL6* (*P* < 0.001) and *COX2* (*P* < 0.001) differed between the experimental treatments in duodenum. Increasing PB inclusion level under heat challenge resulted in linear patterns of decrease for *TLR4* (*P_lin_* < 0.001), *NFKB* (*P_lin_* = 0.023), *TLR3* (*P_lin_* < 0.001), *IRF3* (*P_lin_* = 0.003), *TRIF* (*P_lin_* = 0.009), *TGF* (*P_lin_* = 0.007), *LITAF* (*P_lin_* = 0.005), *iNOS* (*P_lin_* < 0.001), *IL1B* (*P_lin_* < 0.001), *IL6* (*P_lin_* < 0.001), and *COX2* (*P_lin_* < 0.001). Furthermore, *TLR2* (*P_quad_* = 0.011), *TLR3* (*P_quad_* = 0.001), *IRF3* (*P_quad_* = 0.001), *IFNW* (*P_quad_* = 0.015), and *COX2* (*P_quad_* = 0.016) expression displayed quadratic patterns of decrease. Compared to CON, the relative expression levels of *TLR4, TLR3, TGF, iNOS, IL6* and *COX2* were lowest for PB2000 under heat challenge conditions. The expression levels of *LITAF* and *IL1B* were lowest for PB1500 compared to CON and *IRF3* was lowest for PB500 compared to the un-supplemented control treatment. At the liver, the expression of *TLR2* (*P* < 0.001), *TLR4* (*P* = 0.026), *NFKB* (*P* = 0.001), *TLR3* (*P* = 0.001), *IRF3* (*P* = 0.015), *LITAF* (*P* < 0.001), *IL1B* (*P* < 0.001), and *IL6* (*P* < 0.001) differed between the experimental treatments. Increasing PB inclusion level under heat challenge resulted in linear patterns of decrease for *TLR2* (*P_lin_* < 0.001), *TLR4* (*P_lin_* = 0.006), *NFKB* (*P_lin_* < 0.001), *TLR3* (*P_lin_* < 0.001), *IRF3* (*P_lin_* = 0.004), *TRIF* (*P_lin_* = 0.021), *LITAF* (*P_lin_* < 0.001), *IL1B* (*P_lin_* < 0.001), *IL6* (*P_lin_* <0.001), and *IL8* (*P_lin_* = 0.015). The expression of *NFKB* (*P_quad_* = 0.046) and *IL1B* (*P_quad_* = 0.019) displayed quadratic patterns of decrease. Compared to CON, the relative expression levels of *TLR2, TLR4, NFKB, TLR3, IRF3, LITAF, IL1B* and *IL6* were lowest for PB2000. The expression levels of *IL8* was lowest for PB1500 compared to CON and *TRIF* was lowest for PB1500 compared to CON under heat challenge conditions.

**Table 6:** Relative gene expression of inflammation response-related genes in broilers at day 40 of the experiment.

	Genes^4^	Treatments^1^							Statistics^2^		
		CON	PB500	PB1000	PB1500	PB2000	SEM^3^		*P_ANOVA_*	*P_LINEAR_*	*P_QUADRATIC_*
Duodenum	TLR2	1.25	0.89	0.91	0.87	1.17	0.177		0.111	0.675	0.011
	TLR4	2.01^A^	1.29^B^	0.92^BC^	0.82^BC^	0.56^C^	0.218		<0.001	<0.001	0.048
	MYD88	1.25	0.91	1.31	0.99	0.78	0.207		0.076	0.074	0.417
	NFKB1	1.22	0.92	1.17	0.80	0.87	0.154		0.075	0.023	0.770
	TLR3	2.15^A^	1.02^B^	0.85^B^	0.83^B^	0.69^B^	0.212		0.002	<0.001	0.001
	IRF3	1.73^A^	0.76^B^	0.89^B^	1.03^B^	0.94^B^	0.179		<0.001	0.003	0.001
	TRIF	1.43	0.99	0.98	0.96	0.75	0.223		0.106	0.009	0.457
	IFNW	1.14	0.90	0.91	0.87	1.05	0.116		0.119	0.415	0.015
	TGFB1	1.26^A^	0.85^AB^	1.08^AB^	1.00^AB^	0.70^B^	0.152		0.010	0.007	0.844
	LITAF	1.22^a^	1.07^a^	1.17^a^	0.62^b^	0.94^ab^	0.153		0.001	0.005	0.478
	iNOS	1.58^A^	1.15^B^	0.99^BC^	0.86^BC^	0.65^C^	0.109		<0.001	<0.001	0.165
	IL1B	1.59^A^	1.11^AB^	1.03^AB^	0.68^B^	0.80^B^	0.223		0.003	<0.001	0.129
	IL6	1.55^A^	1.01^B^	0.94^B^	0.78^B^	0.68^C^	0.141		<0.001	<0.001	0.042
	IL8	1.43	1.02	1.16	0.82	0.75	0.311		0.221	0.034	0.820
	COX2	1.24^A^	1.20^A^	1.15^A^	0.80^B^	0.61^B^	0.086		<0.001	<0.001	0.016
Liver	TLR2B	1.52^A^	1.14^AB^	1.06^B^	0.91^BC^	0.58^C^		0.141	<0.001	<0.001	0.958
	TLR4	1.28^A^	0.97^AB^	1.00^AB^	1.05^AB^	0.72^B^		0.156	0.026	0.006	0.945
	MYD88	0.93	0.87	1.17	1.16	0.78		0.189	0.171	0.984	0.068
	NFKB1	1.68^A^	1.18^AB^	0.88^B^	0.76^B^	0.73^B^		0.204	0.001	<0.001	0.046
	TLR3	1.45^A^	1.30^AB^	0.86^BC^	0.85^BC^	0.63^C^		0.195	0.001	<0.001	0.582
	IRF3	1.55^a^	0.90^ab^	1.00^ab^	0.84^b^	0.77^b^		0.231	0.015	0.004	0.153
	TRIF	1.17	1.07	0.85	0.98	0.87		0.127	0.087	0.021	0.325
	IFNW	0.96	1.20	0.74	0.92	1.21		0.195	0.130	0.638	0.157
	TGFB1	1.11	1.06	0.89	0.91	0.95		0.134	0.411	0.131	0.299
	LITAF	1.58^A^	1.06^BC^	1.09^B^	0.72^CD^	0.65^D^		0.126	<0.001	<0.001	0.154
	iNOS	1.00	1.00	1.08	0.89	0.87		0.119	0.383	0.169	0.330
	IL1B	1.73^A^	1.00^B^	0.93^B^	0.83^B^	0.74^B^		0.192	<0.001	<0.001	0.019
	IL6	1.44^A^	1.16^AB^	1.01^ABC^	0.75^BC^	0.70^C^		0.155	<0.001	<0.001	0.394
	IL8	1.11	1.17	0.93	0.76	0.78		0.185	0.120	0.015	0.951
	COX2	1.05	0.96	0.96	0.94	0.98		0.150	0.794	0.645	0.556

1 PB Inclusion (CON = 0 mg/kg, PB500 = 500 mg/kg, PB1000 = 1000 mg/kg, PB1500 = 1500 mg/kg, and PB2000 = 2000 mg/kg of diet). Data represent treatment means from n = 7 broiler tissue samples per treatment.

2 Means with different superscripts (a, b, c, d or A, B, C, D) within the same row differ significantly (*P* < 0.05 or 0.01).

3 Standard error of the means.

4 TLR2B = toll-like receptor 2B; TLR4 = toll-like receptor 4; MyD88 = myeloid differentiation primary response 88; NF-κB1 = nuclear factor kappa B subunit 1; TLR3 = toll-like receptor 3; IRF3 = interferon regulatory factor 3; TRIF = toll-like receptor adaptor molecule 1; TGFB1 = transforming growth factor beta 1; LITAF = lipopolysaccharide-induced TNF Factor; iNOS = nitric oxide synthase; IL8 = interleukin 8; IFNW = interferon beta; IL1B = interleukin 1B; IL6 = interleukin 6; COX2 = cyclooxygenase-2.

### MAPK-apoptosis pathway

The relative expression of MAPK-Apoptosis pathway-related genes (*MAPK, FAS, FADD, CHUK, FOS, JUN, CASP3, CASP8* and *BCL2*) at the finisher phase of the experiment in the duodenum and the liver under heat challenge are presented in Table 7. The relative expression of *FAS* (*P* < 0.001), *FADD* (*P* = 0.004), and *FOS* (*P* = 0.017) differed between the experimental treatments in duodenum. Polynomial contrast analysis showed that the expression of *FAS* (*P_lin_* = 0.020), *FADD* (*P_lin_* = 0.004), and *FOS* (*P_lin_* = 0.001) displayed linear patterns of decrease with increasing PB inclusion level. Additionally, *FAS* (*P_quad_* = 0.001) and *FADD* (*P_quad_* = 0.005) expression displayed quadratic patterns of decrease. Compared to the control treatment, the relative expression levels of *FOS, FADD, JUN*, and *FAS* were lowest for PB2000, PB1500, PB1000 and PB500 treatments, respectively. At the liver, the relative expression of *FOS* (*P* = 0.019) differed between the experimental treatments. Polynomial contrast analysis showed that the expression of *FOS* (*P_lin_* = 0.010) displayed linear patterns of decrease with increasing PB inclusion level. Compared to the control treatment, the relative expression of *FOS* was lowest for PB2000 treatment under heat challenge conditions.

**Table 7:** Relative gene expression of apoptosis response-related genes in broilers at day 40 of the experiment.

	Genes^4^	Treatments^1^									Statistics^2^				
		CON	PB500	PB1000	PB1500	PB2000	SEM^3^			*P_ANOVA_*		*P_LINEAR_*		*P_QUADRATIC_*	
Duodenum	MAPK	1.03	0.89	0.95	0.93	0.98		0.092	0.665				0.794		0.237
	FAS	1.44^A^	0.80^B^	1.02^B^	0.87^B^	1.05^B^		0.128	<0.001				0.020		0.001
	FADD	1.29^A^	1.02^AB^	0.96^B^	0.87^B^	1.02^AB^		0.102	0.004				0.004		0.005
	CHUK	1.10	0.97	1.03	0.89	0.96		0.084	0.132				0.061		0.343
	FOS	1.63^a^	1.10^ab^	0.99^ab^	0.84^b^	0.66^b^		0.261	0.017				0.001		0.352
	JUN	1.06	0.97	0.89	0.91	1.15		0.123	0.229				0.708		0.029
	CASP3	1.13	0.95	1.16	0.90	0.93		0.144	0.257				0.181		0.885
	CASP8	1.14	0.91	0.88	1.13	1.02		0.190	0.546				0.981		0.327
	BCL2	1.08	0.99	1.11	0.95	0.83		0.161	0.437				0.145		0.425
Liver	MAPK	1.07	0.88	0.94	0.99	1.02		0.119	0.571				0.923		0.194
	FAS	1.10	1.07	0.81	0.92	0.91		0.135	0.204				0.090		0.255
	FADD	0.91	1.02	0.82	1.12	1.04		0.104	0.060				0.132		0.701
	CHUK	1.05	0.94	0.93	1.07	0.94		0.088	0.340				0.666		0.610
	FOS	2.44^a^	0.79^b^	0.92^b^	0.98^b^	0.72^b^		0.509	0.019				0.010		0.089
	JUN	1.02	0.93	0.79	1.31	1.31		0.239	0.136				0.084		0.189
	CASP3	1.10	0.99	0.98	1.03	0.80		0.143	0.349				0.095		0.662
	CASP8	1.18	0.89	0.85	1.02	1.05		0.143	0.173				0.739		0.032
	BCL2	0.93	1.13	0.95	1.04	1.15		0.166	0.586				0.346		0.824

1 PB Inclusion (CON = 0 mg/kg, PB500 = 500 mg/kg, PB1000 = 1000 mg/kg, PB1500 = 1500 mg/kg, and PB2000 = 2000 mg/kg of diet). Data represent treatment means from n = 7 replicates per treatment.

2 Means with different superscripts (a, b, c or A, B, C) within the same row differ significantly (*P* < 0.05).

3 Standard error of the means.

4 CHUK = conserved helix-loop-helix ubiquitous kinase; FOS = Fos proto-oncogene, AP-1 transcription factor subunit; JUN = Jun proto-oncogene AP-1 transcription factor subunit; FAS/APO-1 = Fas cell surface death receptor; MAPK9 = mitogen-activated protein kinase 9; CASP3,8 = caspase 3,8; FADD = FAS-associated death domain protein; Bc-l2 = *B*-cell lymphoma 2.

## Discussion

Heat stress is one of the most significant stressors in poultry production (Goel, 2021). The present study evaluated the effects of dietary inclusion levels of a phytogenic blend (PB) on broilers’ performance, heat shock, detoxification, antioxidant capacity, inflammation and apoptosis responses at gene transcriptomic level after cyclic heat stress.

Phytogenics demonstrate beneficial effects on the zootechnical performance in poultry (Mountzouris et al., 2011; Hafeez et al., 2016; Greene et al., 2021; Anagnostopoulos et al., 2023; Brouklogiannis et al., 2023). Furthermore, it has been shown that phytogenic effects were dependent on phytogenic composition and inclusion level with (Paraskeuas et al., 2016; Armanini et al., 2021) or without a stressor challenge (Amad et al., 2011).

This study followed a PB dose response to identify the most optimal inclusion level with respect to zootechnical performance and critical biological responses for homeostasis. Broilers administered PB under cyclic heat challenge conditions had improved overall FCR, PER, EER and NEF compared with the un-supplemented control treatment birds. In particular, the performance responses above changed mostly in a quadratic manner with PB inclusion level, highlighting PB1000 and PB1500 as the best PB inclusion levels. Moreover, the PB1000 was the most optimal for overall nutritional efficiency.

Although, currently there can be no direct comparison with other studies, generally, phytogenic applications such as mixtures of carvacrol and thymol (Hashemipour et al., 2013) and encapsulated essential oils dried herbs and saponins (Greene et al., 2021), enhanced broiler FCR in thermoneutral zone and in heat stress conditions, respectively.

In physiological conditions transcription factor *Hsf1* remains bound to *HSP70* and *HSP90* in the cytoplasm (Gouda et al., 2024). Under challenging conditions, cells initiate protective mechanisms to maintain their functional integrity. Specifically, during heat stress, *Hsf1* gets hyperphosphorylated and transforms into a trimer. Subsequently, the *Hsf1* trimer translocate in the nucleus and binds to the heat shock element (HSE) which controls transcription of molecular chaperones that play a critical role in protein folding and result in conglomeration of aggregated or misfolded proteins (Stetler et al., 2010; Ito and Nagata, 2017; Gouda et al., 2024). More specific, *HSP70* binds to newly synthesized proteins to prevent aggregation and assist in proper folding, while *HSP90* aids in the folding of proteins in later stages and alters their conformation. The enhancement of heat shock protein cytoprotective networks is related with maintenance of the master regulator of the homeostasis (Wu et al., 2018; Goel, 2021).

In this work, it was noted that PB inclusion resulted in consistent and beneficial modulation of cellular function by downregulating the expression of the majority of the *Hsf1* pathway genes studied (*HSF1, HSF3, HSF4, HSP27, HSP47, HSP60, HSP70, HSP90* and *HSP100*) in both duodenum (6/9 genes) and liver (8/9 genes), irrespective of PB inclusion level. According to Akbarian et al. (2014), dietary essential oils rich in phenolic compounds suppress HSPs in the liver under heat stress conditions. In addition, several studies have shown that the gut is a significant target for heat stress damage (Quinteiro-Filho et al., 2010; Marchini et al., 2011; Ruff et al., 2020). Given all the above, the noted *Hsf1* pathway modulation by PB inclusion could be considered concomitant with an improved hepatic and duodenal function.

Heat stress leads to an increasing reactive oxygen species (ROS) formation, and causes tissue injury (Tang et al., 2022). In response cells may activate expression of cytoprotective enzymes with detoxifying (e.g., *CYPs, GSTA2*), antioxidant (e.g., *CAT, SOD1, GPX2, GPX7*) and anti-inflammatory (e.g., *HMOX1*) functions (Griela et al., 2021; Brouklogiannis et al., 2023). In particular, Phase I (Ahr pathway) enzymes participate in the oxidative metabolism via their synergies with Phase II enzymes (Nrf2 pathway) (Mountzouris et al., 2022; Brouklogiannis and Mountzouris, 2025). Under homeostatic conditions, *Nrf2* binds to the Kelch-like ECH associating protein 1 (*Keap1*) keeping negatively regulated in the cytoplasm. Potential inducers such us phytogenics cause the disruption of *Nrf2* and *Keap1* cytoplasmic complex and allow *Nrf2* to translocate into the nucleus (Sahin et al., 2013; Seymour et al., 2013). In nucleus, *Nrf2* binds to antioxidant response element (ARE) and regulates the transcription of multiple cytoprotective genes (Ahmed et al., 2017; Brouklogiannis et al., 2023). The AHR–Nrf2 interaction promotes detoxification by synergistically activating, Phase I and II xenobiotic-metabolizing enzymes (XMEs) (Köhle and Bock, 2006).

In this study, the PB inclusion increased the hepatic and duodenal expression of Nrf2 metabolic pathway while decreasing the expression of the Ahr pathway-related genes. Specifically, regarding the Nrf2 pathway 6 out of 9 genes (Phase II enzymes) studied were significantly upregulated both in the duodenum and liver, mostly in a linear manner by PB inclusion. Furthermore, regarding the Ahr pathway 3 out of 5 genes in the duodenum and 4 out of 5 genes (Phase I enzymes) in the liver were down-regulated by PB inclusion. Scientific evidence shows that phytogenic feed additives could provide cytoprotective support under heat stress conditions, maintaining homeostatic balance against ROS in the cell (Akbarian et al., 2014; Liu et al., 2014; Tang et al., 2022). In this study, the pattern of induced adaptive cytoprotection at the duodenum and the liver by PB dietary inclusion level combined with the respective improvements in broiler performance, highlight the beneficial PB role in homeostasis maintenance under thermally challenged conditions.

Toll-like receptors (*TLRs*) play a pivotal role in innate immune system. More specifically, *TLRs* recognize microbial components, metabolites and excess nutrients. The later trigger the activation of transcriptions factors such as nuclear factor-kappa B (*NF-κB*) (Kawai and Akira, 2007; Keestra et al., 2013). *NF-κΒ* controls the transcription of pro-inflammatory (e.g., IL-6, TNF-α) and anti-inflammatory (e.g., *TGF-β*) genes. MAPK signaling pathway participates in the regulation of cell growth, development, and differentiation and plays a significant role in cell multiplication, differentiation, apoptosis, and autophagy (Liu et al., 2024). Furthermore, TLRs trigger the activation of MAPK pathway (Gupta and Knowlton, 2005). In particular, *TLR2* and *TLR4* through *MyD88* initiate the signaling pathways utilizing the *NF-κB* and MAPKs. The encoded enzymes stimulate *NF-κB/AP-1* dependent expression of *Fos/Jun* and the production of proinflammatory cytokines (Griela and Mountzouris, 2023).

In this work, it was noted that PB inclusion down-regulated the expression of the majority of the TLR signaling to NF-κΒ pathway genes in both the duodenum (9 out of 15 genes) and the liver (9 out of 15 genes). Similarly, phytogenic bioactive components modulated TLRs signaling and reduced NF-κΒ activation (Paraskeuas and Mountzouris, 2019). Regarding the MAPK pathway, the dietary PB inclusion had lesser effect on the genes studied as it down-regulated 33 % of the genes at the duodenum and 11 % at the liver. Additionally, the suppressed TLRs signaling - NF-κΒ activation may also be augmented from the up-regulation of the Nrf2 pathway, which is known to have anti-inflammatory activity that inhibit directly or indirectly NF-κB (Sahin et al., 2013). Heat stress may directly interact with TLRs or indirectly enhance the production of HSP70 initiating the inflammatory cascade. Therefore, our findings demonstrate that PB inclusion resulted in a reduced inflammatory level, supporting an overall benefit for homeostatic balance.

Overall, the nutrigenomic analytical data in this work clearly demonstrated an overall PB inclusion down-regulation of mRNA transcripts for heat shock, detoxification, inflammatory and apoptotic related biomarkers, and at the same time an up-regulation of critical adaptive antioxidant defense elements.

In conclusion, the nutrigenomic analytical approach in this work, provided new mechanistic evidence in support of dietary phytogenic benefits for broiler performance, under heat challenge conditions. It was demonstrated that dietary PB inclusion improved broiler performance responses and functioned as a beneficial modulator of critical homeostasis related pathways both at the duodenum and liver under heat challenge conditions. The multi-pathway gene expression patterns determined with PB inclusion were concomitant with improved broiler productive performance under cyclic heat challenge conditions with most of the effects seen at the PB inclusion levels of 1000 to 1500 mg/kg diet in terms of overall nutritional efficiency.

## Declaration of competing interest

The authors declare the following financial interests/personal relationships which may be considered as potential competing interests:

Konstantinos Mountzouris reports financial support was provided by NUEVO SA. If there are other authors, they declare that they have no known competing financial interests or personal relationships that could have appeared to influence the work reported in this paper.
